# Future epidemiological and economic impacts of universal influenza vaccines

**DOI:** 10.1073/pnas.1909613116

**Published:** 2019-09-23

**Authors:** Pratha Sah, Jorge A. Alfaro-Murillo, Meagan C. Fitzpatrick, Kathleen M. Neuzil, Lauren A. Meyers, Burton H. Singer, Alison P. Galvani

**Affiliations:** ^a^Center for Infectious Disease Modeling and Analysis, Yale School of Public Health, New Haven, CT 06520;; ^b^Center for Vaccine Development and Global Health, University of Maryland School of Medicine, Baltimore, MD 21201;; ^c^Department of Integrative Biology, University of Texas at Austin, Austin, TX 78712;; ^d^Emerging Pathogens Institute, University of Florida, Gainesville, FL 32610

**Keywords:** seasonal vaccine, mathematical model, medical cost

## Abstract

The efficacy of influenza vaccines, currently at 44%, is limited by the rapid antigenic evolution of the virus and a manufacturing process that can lead to vaccine mismatch. The National Institute of Allergy and Infectious Diseases (NIAID) recently identified the development of a universal influenza vaccine with an efficacy of at least 75% as a high scientific priority. The US Congress approved $130 million funding for the 2019 fiscal year to support the development of a universal vaccine, and another $1 billion over 5 y has been proposed in the Flu Vaccine Act. Using a model of influenza transmission, we evaluated the population-level impacts of universal influenza vaccines distributed according to empirical age-specific coverage at multiple scales in the United States. We estimate that replacing just 10% of typical seasonal vaccines with 75% efficacious universal vaccines would avert ∼5.3 million cases, 81,000 hospitalizations, and 6,300 influenza-related deaths per year. This would prevent over $1.1 billion in direct health care costs compared to a typical season, based on average data from the 2010–11 to 2018–19 seasons. A complete replacement of seasonal vaccines with universal vaccines is projected to prevent 17 million cases, 251,000 hospitalizations, 19,500 deaths, and $3.5 billion in direct health care costs. States with high per-hospitalization medical expenses along with a large proportion of elderly residents are expected to receive the maximum economic benefit. Replacing even a fraction of seasonal vaccines with universal vaccines justifies the substantial cost of vaccine development.

## Significance

Diminished efficacy of influenza vaccines has fueled research and funding for a broadly protective vaccine. NIAID recently proposed at least 75% efficacy against symptomatic influenza as a key criterion for a universal vaccine. Our analyses demonstrate that universal vaccines with 75% efficacy would be highly impactful in reducing the epidemiological impacts of seasonal influenza at both the national and state levels. Reduced incidence and hospitalizations due to universal vaccine distribution would save $3.5 billion influenza-related direct medical costs per year. This economic benefit surpasses the current and proposed funding of $330 million combined toward the development of a universal influenza vaccine. Our results highlight that benefits of universal vaccine rollout justify the significant investment required for development.

Influenza is responsible for considerable morbidity and mortality worldwide, including an estimated 291,000 to 646,000 deaths annually (1). In the United States, an average of 28.41 million cases, 461,111 hospitalizations, and 40,500 influenza-related deaths occurred each year over the last 9 y (2). The economic burden of influenza has been estimated at $5.8 billion annually, accounting for 65% of the burden from all vaccine-preventable diseases in the United States (3). While vaccination is the primary intervention for influenza prevention and control, the efficacy of the seasonal vaccine has ranged from 19 to 60% during this same time period. Consequently, the National Institute of Allergy and Infectious Disease (NIAID) has identified the development of a more efficacious universal influenza vaccine as a high priority. In concert with the prioritization by NIAID, the US Congress recently approved $130 million for the 2019 fiscal year to support the development of a universal vaccine (4). Another $1 billion over 5 y has been proposed in the Flu Vaccine Act, which is currently under congressional deliberation (5). Similarly, the World Health Organization is advocating for the prioritization of universal influenza vaccine development, and several countries are investing substantially in this research (6, 7).

Seasonal vaccines target the continually evolving globular head of hemagglutinin (HA). Their efficacy, therefore, depends on a close match between the antigens included in the vaccine and those presented by circulating influenza strains. Seasonal vaccine antigens are reformulated annually based on forecasts informed by viral surveillance in over 100 countries. To give manufacturers sufficient lead time to produce enough vaccine doses using the traditional egg-based process, decisions about the antigenic composition of the Northern Hemisphere vaccine are finalized by March each year (8). However, the ensuing 6-mo delay between vaccine recommendation and the influenza season increases the likelihood that the circulating strains will differ from those predicted. The risk of such mismatch is particularly problematic when an antigenic shift, a sporadic event that results in an abrupt major change to the influenza A virus, occurs. Furthermore, viral adaptation to eggs during the manufacturing process can exacerbate the antigenic mismatch between circulating and vaccine strains (9).

A broadly reactive or “universal” vaccine has the potential to overcome the drawbacks of the seasonal vaccine by providing durable protection against all seasonal and pandemic variants of influenza, thereby circumventing the need to reformulate the vaccine each year. Universal vaccines can also be stockpiled to ensure sufficient supply and avoid the shortages that have occurred in the past (10, 11). Development of a broadly protective influenza vaccine, however, has been challenging because of substantial antigenic differences between influenza types and subtypes and an incomplete understanding of protective immunity beyond HA head-based approaches (12, 13). Several novel approaches are being investigated to overcome these hurdles, including targeting more conserved regions of the virus, such as the HA stalk (14) and eliciting cell-mediated immune responses that are more broadly protective (15). In contrast to egg-based manufacturing methods developed in the 1940s, universal vaccines will likely be manufactured using either a cell culture method (16) or a synthetic approach based on reverse genetics (17–19), facilitating rapid production (16). Over 40 additional influenza vaccine candidates are undergoing clinical evaluation (14, 15). A number of promising candidates induce both cell-mediated immunity and humoral immunity against conserved epitopes (20, 21). An intramural NIAID research program is currently conducting clinical trials with a candidate based on the stem of the HA that is conserved among subtypes and is expected to induce broader protection than HA head-based approaches (21, 22). The NIAID is also sponsoring phase III trials of a multimeric candidate that targets conserved regions of the HA, the nucleoprotein, and the M1 protein of the virus (20, 23, 24).

Previous modeling papers have focused on how universal vaccines could impact the evolution of the influenza virus (25) and personal vaccination decisions (26). Yet to be assessed is the epidemiological impact of a universal vaccine in reducing influenza incidence, hospitalizations, and deaths compared with typical seasonal vaccination programs. NIAID has proposed key criteria for a universal influenza vaccine, including at least 75% efficacy against symptomatic influenza and a minimum duration of 1 y (27). To evaluate the epidemiological and societal implications of a universal vaccine that fulfills the NIAID criteria, we developed a transmission model of influenza A subtypes H1N1 and H3N2 and influenza B based on data from the 2010–11 to 2018–19 seasons. Our model takes into account subtype/type- and age-specific differences in transmission, duration of infectiousness, severity, vaccine efficacy, and typical vaccination coverage in each age class. We also incorporated the age-specific burden of comorbidities and their relationship with the severity of clinical outcomes, vaccine uptake, and the robustness of the immune response elicited by vaccination. We quantified the epidemiological and economic impacts of replacing seasonal vaccines either exclusively or partially with universal vaccines. For the state-level analysis, we incorporated state-specific demography, medical costs, and age-specific vaccination uptake. At the national level, our results indicate that replacing even a small fraction of the 169 million vaccine doses currently distributed with universal vaccines could substantially avert incidence, hospitalizations, deaths, and medical costs. Switching entirely to a universal vaccine is projected to reduce the incidence, hospitalizations, and deaths by at least 95%. Our results underscore the enormous economic benefit and public health impact that a universal influenza vaccine could have in the United States and worldwide.

## Results

We calibrated our model of influenza transmission and vaccination to incidence, hospitalizations, deaths, and virologic surveillance data reported by the Centers for Disease Control and Prevention (CDC) from the 2010–11 to 2018–19 seasons (28, 29). Our projection of a typical influenza season in the United States with 169 million doses of 44% efficacious seasonal vaccine results in 17.7 million (95% CI: 17.69 to 17.81 million) cases, 263,429 (95% CI: 262,123 to 264,746) hospitalizations, 20,379 (95% CI: 20,217 to 20,530) deaths, and $3.7 billion (95% CI: 3.68 to 3.72 billion) in direct health care costs. In our calibration, we also calculated vaccine efficacy against hospitalizations and mortality following infection to be 48.1% (95% CI: 47.3 to 48.9%) and 49.9% (95% CI: 49.1 to 50.7%), respectively.

**Fig. 1.**
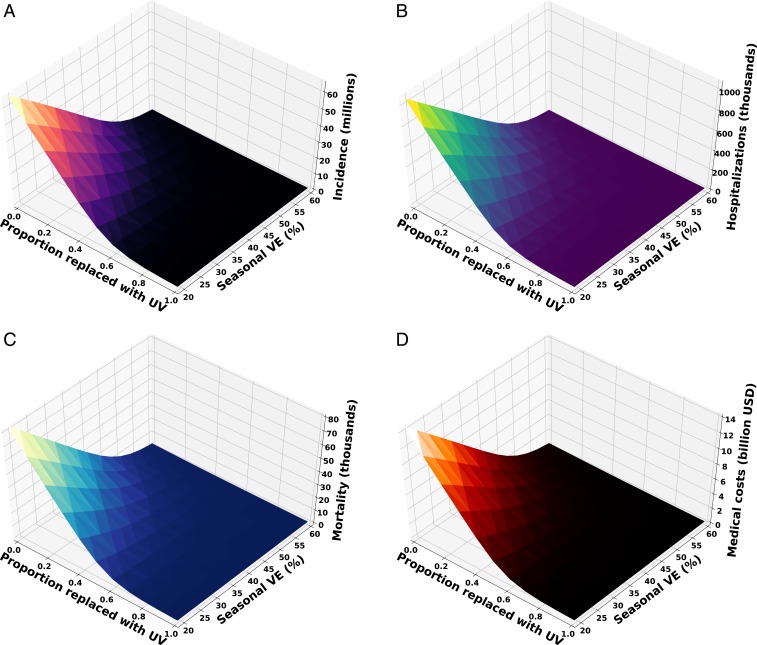
(*A*) Incidence, (*B*) hospitalizations, (*C*) deaths, and (*D*) direct medical costs expected based on both the proportion of seasonal vaccines replaced with universal vaccines (UV) and the seasonal vaccine efficacy (VE).

We then simulated the administration of vaccine doses according to typical age-specific coverage both at the US national level and within each state (30). We compared scenarios in which the age-specific uptake of 169 million currently distributed doses comprises seasonal or universal vaccines distributed exclusively or in combination. We modeled a 75% efficacious universal influenza vaccine, consistent with the NIAID strategic plan, and compared it to seasonal vaccines with efficacies of 19%, 44%, and 60%, corresponding to the minimum, average, and maximum efficacies reported over the 9 seasons spanning 2010–11 to 2018–19 (31). We found that replacing even a small proportion of seasonal vaccine doses with a universal vaccine would be highly impactful in averting infections, hospitalizations, and deaths (Fig. 1). For example, substituting only 10% of typical seasonal vaccines with universal vaccines proportionately across age classes was projected to avert 5.3 million cases, 80,723 hospitalizations, and 6,295 deaths and save $1.13 million in direct medical costs. These health and economic savings would increase as a larger proportion of seasonal vaccines is replaced with universal vaccines. For example, replacing half of the typical seasonal vaccines with universal vaccines would avert 15.1 million cases, 226,823 hospitalizations, and 17,664 deaths and save $3.2 billion in direct medical costs. Complete replacement of seasonal vaccines with universal vaccines would further avert 1.8 million cases, 23,870 hospitalizations, 1,889 deaths, and $341 million in direct medical costs, corresponding to reductions of 96.0, 96.0, 96.1, and 96.0%, respectively, compared with a typical season.

The relative population-level impact of universal vaccines rises when compared to a seasonal vaccine with a lower than typical efficacy, such as the 19% efficacy reported in the years 2014 to 2015. Replacing 50% of these low-efficacy seasonal vaccines with universal vaccines would avert 54.9 million cases, 883,791 hospitalizations, 70,633 deaths, and $12.19 billion in direct medical costs. Complete replacement of less efficacious seasonal vaccines with universal vaccines is projected to further reduce the influenza burden by 11.5 million cases, 168,703 hospitalizations, 13,161 deaths, and $2.37 billion in direct medical costs, corresponding to reductions of more than 98% (Fig. 1).

**Fig. 2.**
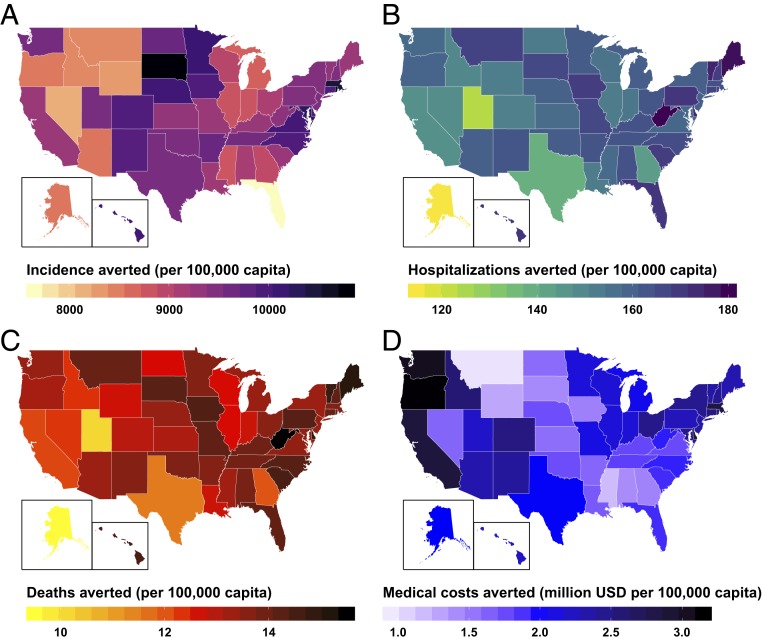
State-level impact per 100,000 capita on (*A*) incidence, (*B*) hospitalizations, (*C*) deaths, and (*D*) direct medical costs averted after replacing 169 million doses of typical 44% efficacious seasonal vaccines with universal vaccines.

We further assessed the impact of universal influenza vaccines within each of the 50 US states based on state-specific demographic composition, typical vaccination coverage, and age-specific vaccine uptake (Fig. 2). We found that universal vaccines would have the greatest impact on reducing incidence for states with high vaccination coverage. For example, replacing seasonal vaccines with universal vaccines would avert the most incidence per capita in South Dakota, Rhode Island, Massachusetts, and Maryland. Vaccination coverage in each of these states exceeds 50% compared with the national average of 45.6%. The most dramatic reduction in severe clinical outcomes is projected for states that have high vaccination coverage as well as a greater proportion of older adults (50+ y). For example, universal vaccines would have the greatest impact on averting hospitalizations and deaths in West Virginia, Maine, and Delaware. In each of these states, vaccination coverage is greater than 47.5%, and older people (50+ y) comprise more than 38% of the population. Nationally, this age class constitutes only 35.4% of the population. Economically, the most substantial savings are projected for Washington, Oregon, and Delaware. These high savings in influenza-related medical costs are driven by the combination of higher costs per hospitalization (32) with a higher proportion of elderly population. Although California has the second-highest hospitalization costs, medical spending per influenza case is lower in this state due to its younger demographic profile.

**Fig. 3.**
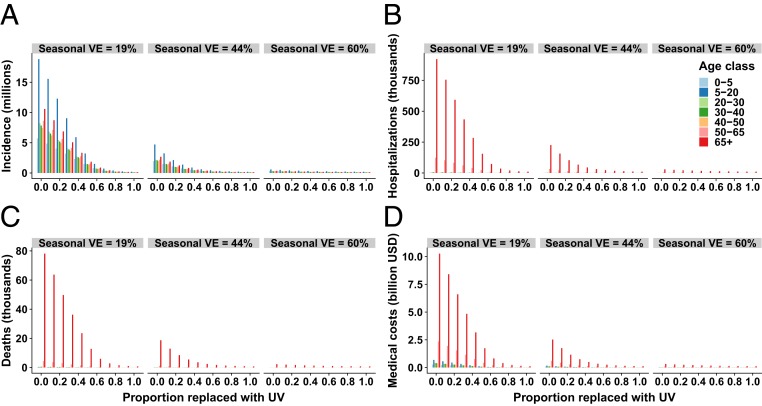
Age-stratified outcomes of (*A*) incidence, (*B*) hospitalizations, (*C*) deaths, and (*D*) direct medical costs expected after replacing (partially or completely) the current seasonal influenza vaccine with a universal influenza vaccine (UV). We present 3 influenza season scenarios with seasonal vaccine efficacies (VE) of 19, 44, and 60%.

Benefits would differ by age class, with universal vaccines projected to be most impactful for reducing incidence within school-age children and for mitigating severe health outcomes and medical costs in the elderly (Fig. 3). Accounting for the current age-specific coverage of seasonal vaccines, school-age children have the highest annual incidence of influenza infections, contributing more than 26.5% of cases despite accounting for only 19% of the population. Replacing half of the typical seasonal vaccines with universal vaccines would avert 4.1 million cases among school-age children. Complete replacement of seasonal vaccines with universal vaccines would further avert 430,000 cases, reducing the incidence rate among school-age children to only 4% of what is expected during a typical influenza season. Compared with other age classes, the elderly have the highest risk of severe health outcomes due to influenza, including hospitalization and death, and therefore also have the highest share of direct medical costs (Fig. 3). Replacing half of the typical seasonal vaccines with universal vaccines would avert 195,131 hospitalizations and 16,231 deaths among this age class, reducing direct medical costs due to these severe medical outcomes by more than 85%. Switching entirely to universal vaccines would almost eliminate influenza transmission, further preventing 20,453 hospitalizations and 1,728 deaths, saving a total of $2.4 billion of direct medical costs in the elderly.

**Fig. 4.**
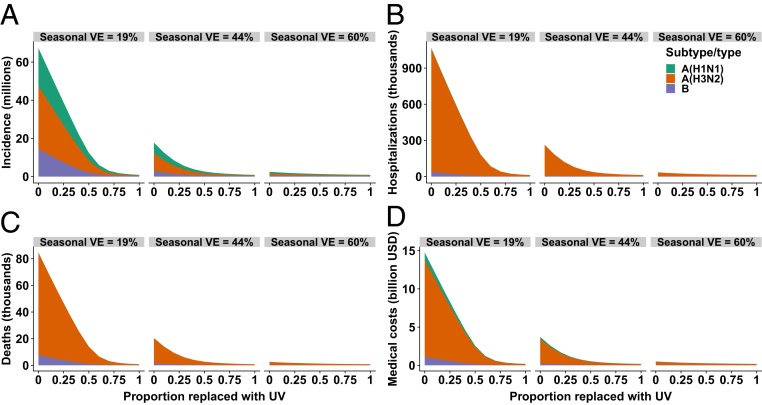
Epidemiological impact of partially or completely replacing seasonal influenza vaccines with universal vaccines (UV) in terms of (*A*) incidence, (*B*) hospitalizations, (*C*) deaths, and (*D*) direct medical costs. We present 3 influenza season scenarios with seasonal vaccine efficacies (VE) of 19, 44, and 60%.

**Fig. 5.**
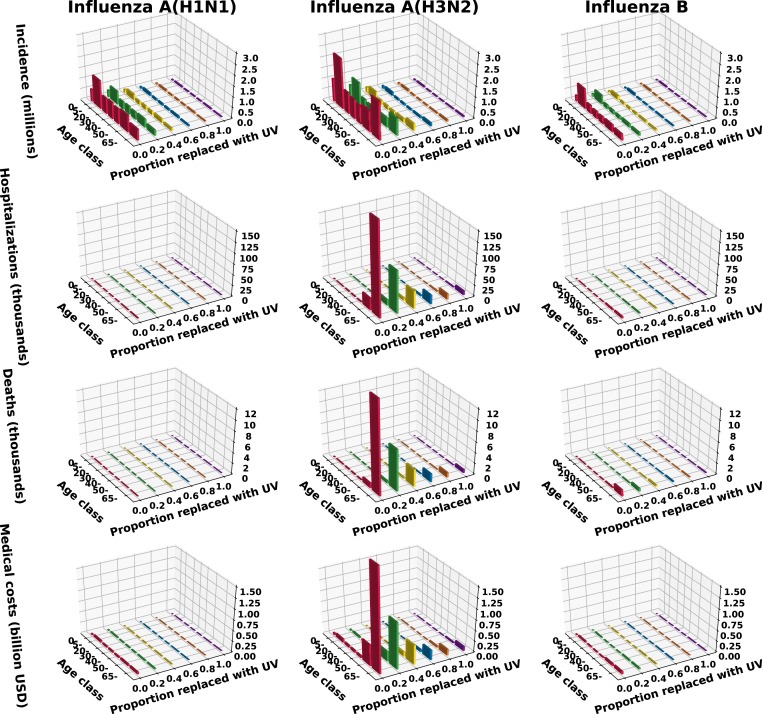
Age- and subtype/type-stratified outcomes of incidence, hospitalizations, deaths, and direct medical costs expected after replacing (partially or completely) the current seasonal influenza vaccine with a universal influenza vaccine. The seasonal vaccine is assumed to be 44% efficacious.

We also considered subtype/type-specific impacts of a universal vaccine with 75% efficacy against influenza A(H1N1), A(H3N2), and B (Fig. 4). These 3 subtypes/types contribute 31.5, 51.5, and 17%, respectively, to the incidence in a typical season where 169 million doses of 44% efficacious vaccines are distributed. We found that even if the efficacy is the same against all influenza subtypes/types, age-specific variation in contact rate combined with the distribution of the subtypes/types among age classes leads to different subtype/type-specific vaccine impacts. For example, replacing half of the typical seasonal vaccines with universal vaccines would reduce influenza A(H1N1), A(H3N2), and B incidence by 84, 87, and 81%, respectively (Fig. 4). Impact on B is lowest because the highest infection rate of this type is reported among school-age children, who are also responsible for most transmission. This vaccination program would avert 3.3 million influenza A and 816,000 influenza B infections among school-age children (Fig. 5), avert 191,680 influenza A and 3,451 influenza B hospitalizations among the elderly, and avert 15,254 influenza A– and 978 influenza B–related deaths among the elderly. Averting these severe health outcomes is projected to save $2.12 billion and $50 million in health care costs in the elderly due to infections from influenza A and influenza B virus, respectively (Fig. 5). Complete replacement of typical seasonal vaccines with universal vaccines is projected to further reduce influenza A(H1N1), A(H3N2), and B incidence to 315,551, 334,085 and 240,941 cases, which would correspond to only 5.6, 3.6, and 8.0% of influenza A(H1N1), A(H3N2), and B incidence observed during a typical seasonal outbreak.

## Discussion

The pandemic threat from viral strain reassortment, the inherent mutability of influenza strains, and the variable effectiveness of seasonal influenza vaccines have galvanized the development of universal influenza vaccines (9, 27). Effectiveness of influenza vaccines in preventing infections has ranged from 19 to 60% over the last 9 seasons, leading to an annual average of 28.41 million cases.

We found that universal vaccines meeting the 75% efficacy goal set by the NIAID would have the potential to avert considerable incidence, hospitalizations, deaths, and economic burden, beyond the protection provided by current seasonal vaccines. At the current age-specific rate of vaccine uptake, switching to universal vaccines is projected to avert 17 million cases, 251,000 hospitalizations, and 19,500 deaths, saving over $3.5 billion in direct medical costs. Given that the capacity to produce 169 million doses of universal vaccines will not immediately exist upon licensure, we considered a range of partial replacement scenarios. Our results indicate that even if universal vaccines only constitute 10% of the doses that are administered in the United States, over 5 million infection cases, 80,000 hospitalizations, 6,000 deaths, and $1 billion in direct medical costs would be averted annually compared to status quo. These results highlight the enormous value of an investment in the research and development of universal influenza vaccines.

At the state level, we found that universal vaccine distribution would be most impactful for reducing influenza incidence in states with high vaccination coverage. Severe clinical outcomes would be most impacted in states that have both an older population and high vaccination coverage. States with a combination of high per-hospitalization medical expenses and a greater proportion of elderly residents are expected to receive the maximum economic benefit from switching to universal vaccines.

Our results show that broadly protective universal vaccines would be highly effective in reducing incidence among all age classes, especially in school-age children. Attenuated immune response to the vaccine in the elderly and an increased risk of influenza complications due to health conditions exacerbate the low efficacy of seasonal vaccines, causing the highest burden of severe complications and medical costs to occur within this age class. Switching to universal vaccines can reduce hospitalization and deaths among the elderly by 95% compared to current levels.

The difficulty of achieving high influenza vaccine uptake (33) has been a persistent public health challenge. Encouragingly, coverage has been increasing over time in all age classes, although it remains below the CDC Healthy People 2020 goals of achieving 80% coverage among people aged 6 mo to 64 y and 90% coverage in those 65 y and over (34). Because of this trend, our projections mimic the age-specific coverage for the most recent season. Accordingly, we project lower morbidity and mortality compared to the average across the seasons for which the model was calibrated. Furthermore, we found that universal vaccines can precipitously reduce influenza transmission without improving the current age-specific vaccination coverages.

In calibrating our model to reported outcomes, we found that the risks of hospitalization and death following infection are substantially lower among vaccinated individuals compared with unvaccinated individuals. This effect is in addition to the reduced risk of infection, as measured by vaccine efficacy. These estimates provide additional evidence that influenza vaccination can reduce disease severity in the event of a breakthrough infection (35).

Our analysis shows the enormous potential economic benefit of universal vaccines. We estimate that the direct medical costs due to influenza exceed $3.7 billion annually, consistent with previous studies (36, 37). Our estimate of the economic burden to society is conservative given that it does not include productivity losses due to illness. These productivity losses could more than double the estimate of economic burden (36, 37). The annual $200 million proposed by the Flu Vaccine Act therefore represent only a fraction of current influenza-related costs. Our estimates of the economic impact of universal vaccines therefore justify the substantial costs of developing a new vaccine (38, 39). In addition, universal vaccines would save time and money spent each year in reformulating current seasonal vaccines. The resulting reduction in revenue stream may disincentivize vaccine manufacturers from fully pursuing innovation toward universal vaccines. Public–private partnerships are therefore critical to support the necessary research and development. Such investment could save the United States money overall while substantially curtailing mortality and morbidity.

One of the key criteria set by NIAID for a universal influenza vaccine is the elicitation of protection which lasts a minimum of 1 y (27). To obtain estimates that were as conservative as possible, we therefore estimated the impact of a universal vaccine over a single influenza season. If the protective effect of universal vaccines extends for multiple seasons, even the current vaccination coverage rate may be sufficient to achieve community protection against seasonal outbreaks of influenza, given the pathogen’s low reproductive number. Further modeling studies are required to more precisely clarify the long-term impact.

Additionally, the potential benefit of a universal influenza vaccine is even greater than estimated here because we did not consider the threat from an influenza pandemic. The World Health Organization identifies an influenza pandemic as one of the top 10 threats to global health in 2019 (40). If a universal influenza vaccine protects against any pandemic influenza strain, that would eliminate delays in the production of a vaccine against the specific pandemic strain and avoid the risks associated with using the seasonal vaccine during a pandemic (41). Even replacing a small proportion of seasonal vaccines with universal vaccines would have a substantial epidemiological and economic impact, underscoring the importance of investing in and accelerating the development of universal influenza vaccines. The savings immediately and over the longer term make the up-front investment in development to be of great societal importance.

## Methods

We modeled the transmission dynamics of influenza A subtypes H1N1 and H3N2, as well as influenza B, in the United States using a system of differential equations. The model stratified the current US demography using census data (42, 43) into 17 age classes: younger than 6 mo, 6 mo to 4 y, 75 y and older in addition to 14 age classes of individuals aged 5 to 74 y old covering 5 y each. Each age class was further stratified into groups with medically low or high risk for influenza complications (44), as well as by vaccination status: unvaccinated, vaccinated with the seasonal vaccine, or vaccinated with a universal vaccine. The influenza epidemic model with vaccination consisted of 714 compartments: for each of the 17 age classes, 3 vaccination groups, and 2 risk groups, there was one susceptible compartment, as well as 3 infectious and 3 recovered compartments corresponding to the 3 viral subtypes/types (*SI Appendix*). Recovered individuals were not susceptible to reinfection by any subtype/type during the remainder of the season. Transmission of each influenza subtype/type within and between age classes depended on the transmissibility of the subtype/type, susceptibility of the age class to that subtype/type, prevalence of infection, vaccination status, and age-specific contact rates. High-risk subgroups had an increased probability of hospitalization and deaths from influenza infection (*SI Appendix*, Table S1). Our model further accounted for subtype/type- and age-specific differences in the infectious period, severity, and vaccine efficacy (*SI Appendix*, Table S1). The probability that a vaccinated individual would be protected from infection depended on baseline vaccine efficacy as well as relative age-specific immunocompetency to mount a protective response. Our model reflected typical vaccination coverage in each age class calculated as an average of coverages reported during 2010–11 to 2018–19 seasons. Direct medical costs included the age-specific cost of an outpatient visit, age-specific cost of hospitalization, and average over-the-counter medication expense for individuals who do not seek medical attention (*SI Appendix*, Table S2). High-risk groups that were not hospitalized had an increased probability of outpatient visits.

Calibration of the influenza transmission and vaccination model was performed using incidence and virologic surveillance data reported by the CDC from the 2010–11 to 2018–19 seasons (28, 29). To calibrate our model, we applied an iterative numerical procedure to find the transmissibility parameter and the age-dependent susceptibility parameters for each influenza subtype/type that best fit the mean incidence and virological profiles (*SI Appendix*, Table S3 and S4). Virologic profiles are positive tests of each subtype/type reported to the CDC based on serological surveillance by public health laboratories located throughout the United States (*SI Appendix*, Table S4). The rate of hospitalization and death given infection, as well as vaccine efficacy in preventing hospitalizations and mortality following infection, were calibrated using the annual mean values of hospitalizations and mortality (*SI Appendix*, Table S5).

We evaluated the impacts of universal vaccine uptake at both national and state levels with regard to 4 outcomes: incidence, hospitalizations, mortality, and direct medical costs. In our results, we present these clinical and economic outcomes consolidated into 7 age classes: 0 to 4, 5 to 19 (school-age children), 20 to 29, 30 to 39, 40 to 49, 50 to 65, and 65 y and older (elderly). For the state-level analysis, we incorporated state-specific demography, medical costs, and age-specific vaccination uptake into simulations. Further methodological detail is provided in the *SI Appendix*.

## Supplementary Material

Supplementary File
