# Supplementary material for: Future epidemiological and economic impacts of universal influenza vaccines
Source: Proc Natl Acad Sci U S A. 2019 Sep 23;116(41):20786–92. doi: 10.1073/pnas.1909613116 (PMC6789917; doi:10.1073/pnas.1909613116)
Supplement: Supplementary File [file pnas.1909613116.sapp.pdf]

Supplementary Information for

Future epidemiological and economic impacts of universal influenza vaccines

Pratha Sah, Jorge A. Alfaro-Murillo, Meagan C. Fitzpatrick, Kathleen M. Neuzil, Lauren A. Meyers, Burton H. Singer, Alison P. Galvani

Burton H. Singer.
E-mail: bhsinger@epi.ufl.edu

This PDF file includes:

Supplementary text
Tables S1 to S6
References for SI reference citations

Supporting Information Text

Model formulation. We considered an epidemic model of transmission with vaccination for three influenza subtypes/types — influenza A(H1N1), influenza A(H3N2) and influenza B. Our compartmental model was stratified by infection status: susceptible (S), infected (I) and recovered (R). We divided the population into seventeen age classes: < 0.5, 0.5–4, 5–9, 10–14, 15–19, 20–24, 25–34, 35–39, 40–44, 45–49, 50–54, 55–60, 60–64, 65–69, 70–74, and 75 years and older. Each age class was further divided into subgroups of individuals with low- (subscript $\downarrow$) and high-risk (subscript $\uparrow$) for influenza related complications. CDC defines high-risk individuals as those with asthma, diabetes, heart conditions, any lung condition other than asthma, kidney condition, obesity, anemia, any neuromuscular condition that makes it difficult to cough, a liver condition or a weakened immune system caused by chronic illness, as well as individuals taking steroids, medicines for chronic illness such as cancer, chemotherapy and HIV/AIDS, and transplant medicines (1). High-risk subgroups had an increased risk of hospitalization and deaths from influenza infections. Each age class was also stratified by vaccination status, including unvaccinated individuals (subscript U), individuals vaccinated with typical seasonal vaccine (subscript T), and individuals vaccinated with the new universal influenza vaccine (subscript N). We assumed that vaccination occurs at the beginning of the influenza season, as recommended by the CDC.

The influenza epidemic model with vaccination consisted of one susceptible compartment, three infectious and three recovered compartments corresponding to the three viral subtypes/types, each for the three vaccination status (unvaccinated, vaccinated with typical seasonal vaccine and vaccinated with universal vaccine) and two risk groups (low-risk and high-risk). We assumed that individuals can get infected only once during a single influenza season. At the beginning of influenza season, the number of susceptible individuals in each category was calculated as:

$$\begin{aligned}
S_{U\downarrow a}(0) &= \mathcal{N}_a(1 - q_{T\cdot\downarrow a} - q_{N\cdot\downarrow a})(1 - q_{\cdot\uparrow a}),\\
S_{U\uparrow a}(0) &= \mathcal{N}_a(1 - q_{T\cdot\uparrow a} - q_{N\cdot\uparrow a})q_{\cdot\uparrow a},\\
S_{T\downarrow a}(0) &= \mathcal{N}_a q_{T\cdot\downarrow a}(1 - q_{\cdot\uparrow a}),\\
S_{T\uparrow a}(0) &= \mathcal{N}_a q_{T\cdot\uparrow a} q_{\cdot\uparrow a},\\
S_{N\downarrow a}(0) &= \mathcal{N}_a q_{N\cdot\downarrow a}(1 - q_{\cdot\uparrow a}),\\
S_{N\uparrow a}(0) &= \mathcal{N}_a q_{N\cdot\uparrow a} q_{\cdot\uparrow a}
\end{aligned} \tag{1}$$

where for each age class a, the proportion of individuals vaccinated with the seasonal vaccine and with the universal vaccine is denoted by $q_{T\cdot a}$ and $q_{N\cdot a}$, respectively. Our model reflected typical vaccination coverage in each age class calculated as an average of coverages reported during 2010-11 to 2018-19 seasons. The proportion of high-risk individuals in each group is represented by $q_{\cdot\uparrow a}$, and $\mathcal{N}_a$ corresponds to the US population for the age class a. Changes within each compartment over time (t) were modeled by the following system of differential equations:

$$\begin{aligned}
\frac{\mathrm{d}}{\mathrm{dt}} S_{U\mathcal{R}a}(t) &= -\sum_i \lambda_a^i(t) S_{U\mathcal{R}a}(t),\\
\frac{\mathrm{d}}{\mathrm{dt}} I^i_{U\mathcal{R}a}(t) &= \lambda_a^i(t) S_{U\mathcal{R}a}(t) - \gamma_a I^i_{U\mathcal{R}a}(t),\\
\frac{\mathrm{d}}{\mathrm{dt}} R^i_{U\mathcal{R}a}(t) &= \gamma_a I^i_{U\mathcal{R}a}(t),\\
\frac{\mathrm{d}}{\mathrm{dt}} S_{W\mathcal{R}a}(t) &= -\sum_i (1 - \epsilon^i_{Wa})\lambda_a^i(t) S_{W\mathcal{R}a}(t),\\
\frac{\mathrm{d}}{\mathrm{dt}} I^i_{W\mathcal{R}a}(t) &= (1 - \epsilon^i_{Wa})\lambda_a^i(t) S_{W\mathcal{R}a}(t) - \gamma_a I^i_{W\mathcal{R}a}(t),\\
\frac{\mathrm{d}}{\mathrm{dt}} R^i_{W\mathcal{R}a}(t) &= \gamma_a I^i_{W\mathcal{R}a}(t),
\end{aligned} \tag{2}$$

where the subscript W represents the vaccine type ($W = T$ for the typical seasonal or $W = N$ for the new universal vaccine), the subscript $\mathcal{R}$ the risk group ($\mathcal{R} =\downarrow$ for low risk or $\mathcal{R} =\uparrow$ for high risk) and the subscript a represents the age class ($a = 1, \ldots, 17$ for ages < 0.5, 0.5–4, 5–9, 10–14, 15–19, 20–24, 25–34, 35–39, 40–44, 45–49, 50–54, 55–60, 60–64, 65–69, 70–74, and 75 years and older, respectively). The recovery rate (γ_a) depended on the age class of the infected individual and influenza subtype/type (Table S1).

To distribute efficacy of vaccine W among age classes that have different immunocompetency to mount a protective response, we assumed that the ratio of age class specific vaccine efficacy to overall vaccine efficacy remains constant. Namely, we assumed that the vaccine efficacy for age class a against vaccine W (ϵ_{Wa}) was dependent on the overall vaccine efficacy (k_W), and an age-dependent immunocompetency to mount a protective response after vaccination (θ_a):

$$\epsilon_{Wa} = \frac{\kappa_W \theta_a}{\kappa'},$$

where κ' represents an overall age-adjusted vaccine efficacy:

$$\kappa' = \sum_a \frac{\mathcal{N}_a}{\sum_b \mathcal{N}_b}\theta_a.$$

For the influenza subtype/type i, the force of infection within each age class was calculated as:

$$\begin{aligned}\lambda_a^i &= \sum_{\alpha=1}^{17}\sum_{\mathcal{R}=\downarrow,\uparrow}\sum_{W=U,T,N}\frac{\beta^i\sigma_a^i\phi_{a\alpha}I^i_{W\mathcal{R}a}}{\mathcal{N}_a},\\ &= \beta_i\sigma_a^i\sum_{\alpha=1}^{17}\sum_{\mathcal{R}=\downarrow,\uparrow}\sum_{W=U,T,N}\frac{\phi_{a\alpha}I^i_{W\mathcal{R}a}}{\mathcal{N}_a}\end{aligned} \qquad [3]$$

where $\phi_{a\alpha}$ is the contact rate for individuals of age class α with individuals of age class a; β^i is the probability of transmission of subtype/type i given a contact of infectious individual with an unvaccinated individual; and σ_a^i is the susceptibility of people in age class a towards subtype/type i.

We parameterized contacts between and within age classes ($\phi_{a\alpha}$ for age classes a and α) using empirical data (2). Epidemiological, immunological and clinical parameters were derived from empirical distributions (Table S1).

Modeling clinical outcomes of hospitalizations and deaths. We took into account age-specific risks of the events (superscript E) of hospitalization ($\mathrm{E}=\mathrm{H}$) and of death ($\mathrm{E}=\mathrm{D}$) for the three influenza subtypes/types. Probability of hospitalization and death for each age class was first calculated as:

$$\mathrm{P}^{\cdot\mathrm{E}}_{\cdot\cdot a} = \rho_{\mathrm{E}}\mathrm{R}^{\cdot\mathrm{E}}_{\cdot\cdot a}, \qquad [4]$$

where ρ_{H} and ρ_{D} are the rates of hospitalization and death given infection, and $\mathrm{R}^{\cdot\mathrm{H}}_{\cdot\cdot a}$ and $\mathrm{R}^{\cdot\mathrm{D}}_{\cdot\cdot a}$ are the age specific rate of hospitalization and deaths relative to the baseline (Table S1). The parameters ρ_{H} and ρ_{D} were used to calibrate hospitalizations and deaths to the average hospitalizations and deaths reported in the US during the 2010-11 to 2018-19 seasons (see below). We assumed that subtype/type-specific probabilities of hospitalizations and deaths among unvaccinated individuals ($\mathrm{P}^{i\mathrm{H}}_{U\cdot a}$ and $\mathrm{P}^{i\mathrm{D}}_{U\cdot a}$, respectively) followed the same probabilities as that of the overall population:

$$\mathrm{P}^{i\mathrm{E}}_{U\cdot a} = \mathrm{R}^{i\mathrm{E}}_{\cdot\cdot a}\frac{\mathrm{P}^{\cdot\mathrm{E}}_{\cdot\cdot a}}{\sum_j \iota^j\mathrm{R}^{j\mathrm{E}}_{\cdot\cdot a}}, \qquad [5]$$

for the events $\mathrm{E}=\mathrm{H},\mathrm{D}$ (hospitalization and death, respectively), where ι^j is the proportion of infections due to subtype/type j and $\mathrm{R}^{j\mathrm{E}}_{\cdot\cdot a}$ is the relative rates of event E for subtype/type j in age class a compared with influenza B (Table S1).

In addition to protecting against infection, vaccination was assumed to have a specific efficacy against hospitalizations ($\epsilon^{i\mathrm{H}}_{V\cdot a}$) and against mortality ($\epsilon^{i\mathrm{D}}_{V\cdot a}$) that was independent of the type of vaccine. We calculated the age- and subtype/type-specific probabilities for vaccinated individuals as:

$$\mathrm{P}^{i\mathrm{E}}_{V\cdot a} = (1-\epsilon^{i\mathrm{E}}_{V\cdot a})\mathrm{P}^{i\mathrm{E}}_{U\cdot a}, \qquad [6]$$

for the events $\mathrm{E}=\mathrm{H},\mathrm{D}$ (hospitalization and death, respectively).

The probability that a vaccinated person of age class a would be protected from hospitalization and death caused by subtype/type i when the vaccine does not take depended on the baseline vaccine efficacy against hospitalizations (δ^{H}) and deaths (δ^{D}). Relative age-specific vaccine efficacy in preventing hospitalizations and deaths compared to baseline is denoted $\Delta_a^{i\mathrm{H}}$ and $\Delta_a^{i\mathrm{D}}$, respectively. Thus, for each group, the efficacy of vaccines in preventing an event of hospitalization ($\mathrm{E}=\mathrm{H}$) or death ($\mathrm{E}=\mathrm{D}$) among vaccinated people for whom the vaccine does not take was calculated as $\epsilon^{i\mathrm{E}}_{V\cdot a}=\delta^{\mathrm{E}}\Delta_a^{i\mathrm{E}}$.

We assumed that hospitalization and mortality rate among high-risk ($\mathcal{R}=\uparrow$) vaccinated (V) and unvaccinated (U) individuals followed the same probability as the overall population. The rates were therefore calculated as:

$$\begin{aligned}\mathrm{R}^{i\mathrm{E}}_{U\uparrow a} &= \frac{\mathrm{R}^{i\mathrm{E}}_{\cdot\uparrow a}}{\iota_U + (1-\epsilon^{i\mathrm{E}}_{V\cdot a})\iota_V},\\ \mathrm{R}^{i\mathrm{E}}_{V\uparrow a} &= (1-\epsilon^{i\mathrm{E}}_{V\cdot a})\mathrm{R}^{i\mathrm{E}}_{U\uparrow a},\end{aligned} \qquad [7]$$

for the events hospitalization ($\mathrm{E}=\mathrm{H}$) and death ($\mathrm{E}=\mathrm{D}$), where ι_U and ι_V are the proportion of infected individuals that unvaccinated and vaccinated, respectively. The parameters $\mathrm{R}^{i\mathrm{H}}_{\cdot\uparrow a}$ and $\mathrm{R}^{i\mathrm{D}}_{\cdot\uparrow a}$ are the relative risk of hospitalizations and deaths for high-risk individuals due to infection from subtype/type i which were derived from the empirical literature (Table S1).

The number of cumulative hospitalizations ($E=H$) and deaths ($E=D$) for unvaccinated (U) and vaccinated (V) in each risk group (i.e., low risk group $\mathcal{R}=\downarrow$ and high risk group $\mathcal{R}=\uparrow$) and age class were then calculated as:

$$\begin{aligned}E^i_{U\downarrow a} &= C^i_{U\downarrow a}\mathrm{P}^{i\mathrm{E}}_{U\cdot a},\\ E^i_{U\uparrow a} &= C^i_{U\uparrow a}\mathrm{R}^{i\mathrm{E}}_{U\uparrow a}\mathrm{P}^{i\mathrm{E}}_{U\cdot a},\\ E^i_{V\downarrow a} &= C^i_{V\downarrow a}\mathrm{P}^{i\mathrm{E}}_{V\cdot a},\\ E^i_{V\uparrow a} &= C^i_{V\uparrow a}\mathrm{R}^{i\mathrm{E}}_{V\uparrow a}\mathrm{P}^{i\mathrm{E}}_{V\cdot a},\end{aligned} \qquad [8]$$

where $C^i_{U\mathcal{R}a}$ and $C^i_{V\mathcal{R}a}$ represent the cumulative number of infections by subtype/type i, for individuals of risk group $\mathcal{R}$ and age class a that were unvaccinated and vaccinated, respectively.

Quantifying direct health care cost of influenza

We took into account age-specific direct health care cost of influenza. Direct medical expense included age-specific cost of outpatient visit and average over-the-counter medications for individuals that do not seek medical attention. Each infection case had a probability of outpatient visit given influenza. Each outpatient visit and hospitalization case was associated with an age-specific cost. The probability that an individual with influenza would not seek medical attention, was assumed to equal one minus the sum of the probabilities of outpatient visit, hospitalization and death given influenza infection. Table S2 shows all costs and probabilities used to quantify the direct health care cost of influenza.

Model calibration

We parameterized our model of influenza transmission and vaccination with a distribution of values for the epidemiological, immunological and clinical parameters from the empirical published literature (Table S1). Model calibration was performed using the dataset on incidence, hospitalizations, death and age-specific virologic surveillance from nine influenza seasons (2010–11 to 2018–19) reported by CDC (Table S3, S4). Each input parameter distribution and data was sampled 5,000 times. We used a two-step procedure to calibrate our model. First, an optimization procedure was used to find the transmission parameters for each influenza subtypes/types (β_{H1N1}, β_{H3N2}, β_B) and the age-specific susceptibility to the three viral subtypes/types that best fitted the average incidence and virologic profile across the 5,000 sampled parameter set. In the next step, the same optimization procedure was used to fit four parameters— vaccine efficacy against hospitalizations (δ^H) and deaths (δ^D) given an infection, as well as the hospitalization (ρ_H) and death (ρ_D) rate given infection—to the average hospitalization and death data reported in the US. The optimization was performed using a truncated Newton algorithm implemented in the the Python package SciPy (http://www.scipy.org/).

State level impact of vaccination

We incorporated state-specific demography, medical costs, and age-specific vaccination uptake in our state-specific simulations (Table S6). Since state-level data of virologic surveillance was unavailable, we assumed state specific epidemiological parameters were same as those obtained in the model calibration. Out of the 169 million doses distributed in US, we assumed total doses distributed in each state followed the same ratio as the state-level vaccination coverage reported by CDC in May, 2019 (3). The total doses allocated to each state were further distributed among different age classes based on average age-specific vaccine update data reported in each state during 2010–11 to 2018–19 seasons (Table S6). Direct medical costs in each state were adjusted using data of average health care expense per capita by state (Table S6, (4)).

Table S1. Distribution of epidemiological, immunological and clinical parameters from the literature used in our model

Parameter	age class	Influenza A(H1N1)	Influenza A(H3N2)	Influenza B	Reference
Infectious period (days), $\frac{1}{\gamma_a}$	0-14	T(2.3, 3.6, 5.2)	T(2.3, 3.6, 5.2)	T(2.3, 3.6, 5.2)	(5)
	14+	T(3.2, 3.9, 4.9)	T(3.2, 3.9, 4.9)	T(3.2, 3.9, 4.9)	(5)
Immunocompetency to mount a protective response after vaccination, θ_a	0.5-4	T(0.50, 0.60, 0.68)	T(0.50, 0.60, 0.68)	T(0.50, 0.60, 0.68)	(6)
	5-17	T(0.35, 0.46, 0.56)	T(0.35, 0.46, 0.56))	T(0.35, 0.46, 0.56)	(6)
	18-49	T(0.26, 0.39, 0.50)	T(0.26, 0.39, 0.50)	T(0.26, 0.39, 0.50)	(6)
	49+	T(0.08, 0.33, 0.51)	T(0.08, 0.33, 0.51)	T(0.08, 0.33, 0.51)	(6)
Rate of hospitalization given an infection, $\mathrm{R}^{\cdot\mathrm{H}}_{\cdot\cdot a}$ (percentage)	0-4	T(0.49, 1.41, 2.33)	T(0.49, 1.41, 2.33)	T(0.49, 1.41, 2.33)	(7, 8)
	5-17	T(0.02, 0.06, 0.1)	T(0.02, 0.06, 0.1)	T(0.02, 0.06, 0.1)	(7, 8)
	18-49	T(0.15, 0.42, 0.69)	T(0.15, 0.42, 0.69)	T(0.15, 0.42, 0.69)	(7, 8)
	50-64	T(0.676, 1.93, 3.18)	T(0.676, 1.93, 3.18)	T(0.676, 1.93, 3.18)	(7, 8)
	64+	T(1.47, 4.21, 6.95)	T(1.47, 4.21, 6.95)	T(1.47, 4.21, 6.95)	(7, 8)
Rate of death given an infection, $\mathrm{R}^{\cdot\mathrm{D}}_{\cdot\cdot a}$ (percentage)	0-4	T(0.002, 0.004, 0.006)	T(0.002, 0.004, 0.006)	T(0.002, 0.004, 0.006)	(7)
	5-17	T(0.0008 , 0.001, 0.0012)	T(0.0008 , 0.001, 0.0012)	T(0.0008 , 0.001, 0.0012)	(7)
	18-49	T(0.003, 0.009, 0.015)	T(0.003, 0.009, 0.015)	T(0.003, 0.009, 0.015)	(7)
	50-64	T(0.0458, 0.134, 0.222)	T(0.0458, 0.134, 0.222)	T(0.0458, 0.134, 0.222)	(7)
	64+	T(0.406, 0.117, 0.193)	T(0.406, 0.117, 0.193)	T(0.406, 0.117, 0.193)	(7)
Relative hospitalization rate compared to influenza B, $\mathrm{R}^{j\mathrm{H}}_{\cdot\cdot a}$	0-4	T(0,8,23)/T(2,41,86)	T(1,61,124)/T(2,41,86)	1	(9)
	5-17	T(0,3,9)/T(0,8,15)	T(0,8,17)/T(0,8,15)	1	(9)
	18-49	T(0,1,3)/T(0,6,11)	T(0,10,20)/T(0,6,11)	1	(9)
	50-64	T(0,1,1)/T(0,7,15)	T(0,17,35)/T(0,7,15)	1	(9)
	65-74	0	T(1,39,80)/T(0,9,18)	1	(9)
	74+	0	T(3,117,235)/T(1,40,80)	1	(9)
Relative death rate compared to influenza B, $\mathrm{R}^{j\mathrm{D}}_{\cdot\cdot a}$	0-4	0.444	1.333	1	(10)
	5-17	0.5882	0.8823	1	(10)
	18-49	0.1245	1.5897	1	(10)
	50-64	0.0277	2.7944	1	(10)
	65-74	0.0016	3.1693	1	(10)
	74+	0	1.4822	1	(10)
Relative rate of hospitalization for high risk individuals compared to low risk individuals, $\mathrm{R}^{i\mathrm{H}}_{\cdot\uparrow a}$	0-4	0	T(0, 11, 26)/T(1, 61, 124)	T(0, 10, 14)/T(2, 41, 86)	(9)
	5-17	T(0, 3, 9)/T(0, 3, 9)	T(0, 4, 8)/T(0, 8, 17)	T(0, 3, 7)/T(0, 8, 15)	(9)
	18-49	T(0, 9, 30)/T(0, 1, 3)	T(1, 52, 110)/T(0, 10, 20)	T(1, 30, 64)/T(0, 6, 11)	(9)
	50-64	T(0, 10, 33)/T(0, 1, 1)	T(3, 149, 313)/T(0, 17, 35)	T(2, 60, 134)/T(0, 7, 15)	(9)
	65-74	0	T(6, 286, 591)/T(1, 39, 80)	T(1, 44, 105)/T(0, 9, 18)	(9)
	74+	T(0, 21, 86)/T(0, 2, 5)	T(12, 587, 1198)/T(3, 117, 235)	T(4, 161, 380)/T(1, 40, 80)	(9)
Relative death rate for high risk individuals compared to low risk individuals, $\mathrm{R}^{i\mathrm{D}}_{\cdot\uparrow a}$	0-4	0.454	0.333	0.44	(10)
	5-17	0.667	0.579	2.778	(10)
	18-49	1.267	1.568	1.294	(10)
	50-64	12	3.979	3.047	(10)
	65-74	1	5.813	5.945	(10)
	74+	1	2.659	2.998	(10)
Relative age-specific vaccine efficacy against hospitalization given an infection compared to baseline (δ^H), $\Delta^{i\mathrm{H}}_{V\cdot a}$ (percentage)	0.5-15	T(27.3, 82.1, 95.6)	T(27.3, 82.1, 95.6)	T(27.3, 82.1, 95.6)	(11)
	16-64	T(34, 55, 76)	T(38, 50, 62)	T(8, 45, 81)	(12)
	64+	T(26, 54, 82)	T(21, 33, 45)	T(11, 31, 51)	(12)
Relative age-specific vaccine efficacy against death an given infection compared to baseline (δ^D), $\Delta^{i\mathrm{D}}_{V\cdot a}$ (percentage)	0.5-17	T(31, 59, 77)	T(31, 59, 77)	T(43, 71, 87)	(13)
	18-64	U(70, 90)	U(70, 90)	U(70, 90)	(14)
	64+	T(11, 21, 29)	T(11, 21, 29)	T(11, 21, 29)	(15)

T = Triangular distribution with parameters minimum value, mode, and maximum value.
U = Uniform distribution with parameters minimum and maximum value.

Table S2. Parameters used to quantify direct health care cost of influenza

Parameter	Age class	Distribution	Reference
Probability of outpatient visit given influenza for low-risk individuals	0-4	N(0.455,0.098)	(16)
	5-17	N(0.318,0.061)	(16)
	18-64	N(0.313,0.014)	(16)
	65+	N(0.620,0.027)	(16)
Probability of outpatient visit given influenza for high-risk individuals	0-4	N(0.910,0.250)	(16)
	5-17	N(0.635,0.167)	(16)
	18-64	N(0.625,0.118)	(16)
	65+	N(0.850,0.093)	(16)
Cost of an outpatient visit given influenza (2018 US dollars)^b	< 1	84.74	(17)
	1-17	90.62	(17)
	18-44	114.04	(17)
	45-64	103.96	(17)
	65+	100.16	(17)
Cost of a hospitalization given influenza (2018 US dollars)^b	< 1	6102.51	(17)
	1-17	7370.06	(17)
	18-44	9590.51	(17)
	45-64	15879.94	(17)
	65-84	10595.48	(17)
	85+	9670.29	(17)
Over-the-counter medications without medical attention (2018 US dollars)^b	all	4.11	(7)

N = Normal distribution with parameters mean and variance.
The cost obtained from the reference was adjusted to 2018 US dollars.

Table S3. Vaccine efficacy, vaccine doses distributed, incidence, hospitalizations, and deaths reported in the US from 2010–2011 to 2018–19 seasons.

Year	Vaccine efficacy (%)	Doses (millions)	Incidence (millions)	Hospitalizations (thousands)	Deaths (thousands)
2010-2011	T(53, 60, 66)	155.1	T(20.0, 21.0, 25.0)	T(270, 290, 370)	T(32.0, 37.0, 51.0)
2011-2012	T(36, 47, 56)	132.0	T(8.7, 9.3, 12.0)	T(130, 140, 190)	T(11.0, 12.0, 23.0)
2012-2013	T(43, 49, 55)	134.9	T(32.0, 34.0, 38.0)	T(530, 570, 680)	T(37.0,43.0,57.0)
2013-2014	T(44, 52, 59)	134.5	T(28.0, 30.0, 33.0)	T(320, 350, 390)	T(33.0,38.0,50.0)
2014-2015	T(10, 19, 27)	147.8	T(29.0, 30.0, 33.0)	T(540, 590, 680)	T(44.0,51.0,64.0)
2015-2016	T(41, 48, 55)	146.4	T(24.0, 25.0, 28.0)	T(290, 310, 340)	T(21.0,25.0,31.0))
2016-2017	T(32, 40, 46)	145.9	T(28.0, 30.0, 32.0)	T(520, 580, 660)	T(44.0,51.0,64.0)
2017-2018	T(31, 38, 43)	155.3	T(46.0, 49.0, 53.0)	T(870, 960, 1100)	T(69.0,79.0,99.0)
2018-2019	T(34, 47, 57)	169.1	U(25.5, 29.3)	U(327, 394)	U(21.5, 35.5)

T = Triangular distribution with parameters minimum value, mode, and maximum value.

Table S4. Age-specific virologic surveillance in the US from 2010–2011 to 2018–19. The values are positive tests of each subtype/type reported to the CDC based on serological surveillance by public health laboratories located throughout the US.

Year	Influenza A(H1N1)				Influenza A(H3N2)				Influenza B			
	0-4 yr	5-24 yr	25-64 yr	65+ yr	0-4 yr	5-24 yr	25-64 yr	65+ yr	0-4 yr	5-24 yr	25-64 yr	65+ yr
2010-2011	4.1	13.0	13.1	1.2	8.0	12.1	14.7	11.8	3.6	12.3	4.6	1.4
2011-2012	3.0	7.1	7.9	0.8	11.1	21.9	20.0	14.7	2.0	6.9	3.6	1.1
2012-2013	0.7	1.3	2.3	0.2	8.7	19.4	22.6	22.0	3.6	10.6	6.1	2.6
2013-2014	8.9	18.0	40.5	8.4	1.1	3.3	4.1	3.8	1.1	3.6	4.9	2.3
2014-2015	0.1	0.1	0.2	0.0	7.8	22.8	22.9	32.1	1.3	4.6	5.0	3.0
2015-2016	7.5	13.7	27.8	7.7	1.3	6.1	4.3	3.4	2.5	10.7	9.9	5.0
2016-2017	0.41	0.57	1.09	0.46	6.17	23.0	23.55	28.18	1.0	4.48	5.67	5.42
2017-2018	1.77	3.27	5.19	1.86	4.93	13.56	20.35	26.02	1.31	6.10	7.80	7.84
2018-2019	9.46	18.62	27.17	10.22	3.61	14.46	8.09	7.45	0.1	0.35	0.3	0.18

Table S5. Calibrated parameters

Parameter	age class	Value (95% CI)
Transmission probability of influenza A(H1N1), β^{H1N1}	all	0.00202 (0.002,0.00203)
Transmission probability of influenza A(H3N2), β^{H3N2}	all	0.00290(0.00287, 0.00293)
Transmission probability of influenza B, β^{B}	all	0.00202 (0.00201, 0.00203)
Susceptibility towards influenza A (H1N1), σ^{H1N1}	0-5	0.817 (0.813, 0.822)
	5-25	0.664 (0.660, 0.667)
	25-65	0.684(0.680, 0.688)
	65+	0.774(0.770, 0.779)
Susceptibility towards influenza A (H3N2), σ^{H3N2}	0-5	0.704(0.699, 0.709)
	5-25	0.548(0.542, 0.554)
	25-65	0.440(0.435, 0.446)
	65+	0.956(0.953, 0.959)
Susceptibility towards influenza B, σ^{B}	0-5	0.746(0.741, 0.751)
	5-25	0.735(0.731, 0.739)
	25-65	0.582 (0.577, 0.586)
	65+	0.807 (0.802, 0.812)
Vaccine efficacy against hospitalization, δ^H	all	0.481 (0.473, 0.489)
Vaccine efficacy against death, δ^D	all	0.499 (0.491, 0.507)
Hospitalization rate given infection, ρ_H	all	2.93×10^{-4} (2.88×10^{-4}, 2.98×10^{-4})
Death rate given infection, ρ_D	all	3.74×10^{-5} (3.69×10^{-5}, 3.80×10^{-5})

Table S6. Demographic and economic parameters used for state specific simulations

State	Vaccine doses distributed (millions)	Age-specific vaccine uptake (percentage)						Medical expense relative to national average
		6mo-4yr	5-12yr	13-17 yr	18-49yr	50-64 yr	65+ yr	
Alabama	2.53	66.06	56.36	41.91	30.21	44.66	65.94	0.64
Alaska	0.34	57.56	49.03	35.66	32.21	37.90	53.93	1.22
Arizona	3.26	63.38	52.95	34.50	26.54	39.64	60.90	1.09
Arkansas	1.63	64.21	69.38	52.63	31.03	44.29	62.93	0.73
California	19.84	69.25	60.32	45.01	29.60	41.89	62.83	1.42
Colorado	3.11	73.56	60.63	45.91	35.64	47.70	69.05	1.20
Connecticut	2.08	84.24	69.68	49.26	34.65	46.91	66.96	1.11
Delaware	0.56	74.99	65.60	50.05	34.58	48.21	69.78	1.25
Florida	9.16	59.21	48.30	35.36	22.64	34.86	61.39	0.87
Georgia	4.97	63.29	53.34	38.21	28.74	41.24	62.68	0.74
Hawaii	0.85	72.24	72.79	55.86	36.59	46.63	67.88	0.96
Idaho	0.76	61.72	45.31	32.64	26.74	37.79	58.29	1.29
Illinois	6.25	68.64	53.28	39.18	28.33	41.46	61.55	1.04
Indiana	3.24	62.08	54.31	36.47	28.41	42.49	61.56	1.03
Iowa	1.85	69.99	57.14	40.49	37.70	51.25	72.29	0.64
Kansas	1.53	68.00	53.81	36.54	32.70	44.75	65.14	0.74
Kentucky	2.38	65.48	52.56	41.40	31.85	47.89	69.26	0.80
Louisiana	2.40	65.94	56.81	46.69	30.10	43.61	63.03	0.81
Maine	0.75	69.41	63.03	50.91	32.39	46.84	65.80	0.97
Maryland	3.60	76.70	70.30	51.88	37.69	48.51	68.18	1.13
Massachusetts	4.20	82.75	73.43	61.24	39.29	50.58	65.15	1.24
Michigan	4.89	62.11	54.50	40.26	28.28	41.90	61.80	0.96
Minnesota	3.28	71.79	63.04	45.63	38.70	49.74	69.09	0.96
Mississippi	1.46	56.86	49.39	37.93	29.10	41.14	65.84	0.56
Missouri	3.27	63.58	55.23	38.34	32.63	45.20	70.11	0.94
Montana	0.51	62.55	47.16	33.06	28.64	41.83	63.65	0.44
Nebraska	1.11	73.06	61.82	44.34	36.95	49.76	67.84	0.79
Nevada	1.29	59.43	51.34	35.94	24.66	35.61	57.81	0.82
New Hampshire	0.75	72.01	66.51	48.74	34.66	46.16	64.77	1.07
New Jersey	4.81	82.66	69.55	45.85	29.73	41.95	64.88	1.09
New Mexico	1.16	74.05	66.69	52.09	32.90	44.41	61.49	1.08
New York	10.72	72.04	65.68	48.84	31.65	45.39	64.56	1.13
North Carolina	5.88	69.32	61.19	44.94	35.56	50.11	72.13	0.87
North Dakota	0.42	73.28	62.74	47.06	35.63	45.86	64.35	0.76
Ohio	6.07	66.53	54.21	41.16	31.35	45.15	63.85	1.13
Oklahoma	2.14	63.99	55.26	39.96	32.85	48.59	70.27	0.80
Oregon	1.95	61.53	53.20	39.41	28.14	40.79	58.76	1.48
Pennsylvania	7.10	75.35	63.21	46.88	32.69	45.38	66.54	0.99
Rhode Island	0.68	85.58	79.73	66.98	41.13	51.09	66.46	1.13
South Carolina	2.66	66.35	59.08	39.25	30.50	46.68	68.80	0.84
South Dakota	0.56	73.69	65.76	54.47	44.24	52.38	71.45	0.62
Tennessee	3.66	69.76	59.21	47.14	32.99	47.05	67.30	0.82
Texas	14.36	69.16	60.01	43.93	30.25	43.60	64.56	1.04
Utah	1.55	63.93	53.33	35.21	33.54	44.81	60.74	1.25
Vermont	0.35	72.40	60.20	45.88	33.82	48.01	67.48	1.01
Virginia	4.90	73.80	62.19	45.26	38.00	49.60	67.80	0.81
Washington	4.01	70.40	57.04	42.35	34.69	47.94	66.94	1.41
West Virginia	1.06	63.76	56.05	43.18	34.33	52.46	72.35	0.82
Wisconsin	2.87	69.94	59.33	42.70	30.74	38.88	55.80	1.03
Wyoming	0.27	57.65	45.53	32.41	27.28	39.09	60.14	0.63

References

1. Centers for Disease Control and Prevention (2018) National early season flu vaccination coverage, United States, November 2014 (https://www.cdc.gov/flu/fluvaxview/nifs-estimates-nov2014.htm). Page last updated when retrieved: June 24, 2016.
2. Mossong J, et al. (2008) Social Contacts and Mixing Patterns Relevant to the Spread of Infectious Diseases. *PLoS Medicine* 5(3):e74.
3. Centers for Disease Control and Prevention (2018) 2017-18 influenza season vaccination coverage report (https://www.cdc.gov/flu/fluvaxview/reportshtml/reporti1718/reporti/index.html). Retrieved: January 7, 2019.
4. The Henry J. Kaiser Family Foundation (2014) Health care expenditures per capita by state of residence (https://www.kff.org/other/state-indicator/health-spending-per-capita/). Retrieved: January 7, 2019.
5. Cauchemez S, Carrat F, Viboud C, Valleron AJ, Boëlle PY (2004) A Bayesian MCMC approach to study transmission of influenza: Application to household longitudinal data. *Statistics in Medicine* 23(22):3469–3487.
6. Cowling BJ, Feng S, Finelli L, Steffens A, Fowlkes A (2016) Assessment of influenza vaccine effectiveness in a sentinel surveillance network 2010–13, United States. *Vaccine* 34(1):61–66.
7. Molinari NAM, et al. (2007) The annual impact of seasonal influenza in the US: measuring disease burden and costs. *Vaccine* 25(27):5086–5096.
8. de Boer PT, et al. (2016) Cost-effectiveness of quadrivalent versus trivalent influenza vaccine in the United States. *Value in Health* 19(8):964–975.
9. Matias G, et al. (2017) Estimates of hospitalization attributable to influenza and RSV in the US during 1997–2009, by age and risk status. *BMC public health* 17(1):271.
10. Matias G, et al. (2014) Estimates of mortality attributable to influenza and RSV in the United States during 1997–2009 by influenza type or subtype, age, cause of death, and risk status. *Influenza and other respiratory viruses* 8(5):507–515.
11. Buchan SA, et al. (2017) Vaccine effectiveness against laboratory-confirmed influenza hospitalizations among young children during the 2010-11 to 2013-14 influenza seasons in Ontario, Canada. *PLoS ONE* 12(11):1–15.
12. Rondy M, et al. (2017) Effectiveness of influenza vaccines in preventing severe influenza illness among adults: A systematic review and meta-analysis of test-negative design case-control studies. *Journal of Infection* 75(5):381–394.
13. Flannery B, et al. (2017) Influenza vaccine effectiveness against pediatric deaths: 2010-2014. *Pediatrics* 139(5):e20164244.
14. Miller M, et al. (2008) Prioritization of influenza pandemic vaccination to minimize years of life lost. *The Journal of Infectious Diseases* 198(3):305–311.
15. Mangtani P, et al. (2004) A cohort study of the effectiveness of influenza vaccine in older people, performed using the United Kingdom General Practice Research Database. *The Journal of Infectious Diseases* 190(1):1–10.
16. Lee, Bruce Y, et al. (2012) The potential economic value of a 'universal'(multi-year) influenza vaccine. *Influenza and other respiratory viruses* 6(3):167–175.
17. Lee, Bruce Y, et al. (2015) Quantifying the economic value and quality of life impact of earlier influenza vaccination. *Medical Care* 53(3):218.
